# Supplementary material for: Soft, miniaturized, wireless olfactory interface for virtual reality
Source: Nat Commun. 2023 May 9;14:2297. doi: 10.1038/s41467-023-37678-4 (PMC10169775; doi:10.1038/s41467-023-37678-4)
Supplement: Supplementary file 1 — Supplementary Information [file 41467_2023_37678_MOESM1_ESM.pdf]

Supplementary Information

Soft, Miniaturized, Wireless Olfactory Interface for Virtual Reality

Yiming Liu[1†], Chun Ki Yiu[1,2†], Zhao Zhao[3,4†], Wooyoung Park[1†], Rui Shi[1], Xingcan Huang[1], Yuyang Zeng[1], Kuan Wang[3], Tsz Hung Wong[1], Shengxin Jia[1,2], Jingkun Zhou[1,2], Zhan Gao[1], Ling Zhao[1], Kuanming Yao[1], Jian Li[1,2], Chuanlu Sha[1], Yuyu Gao[1,2], Guangyao Zhao[1], Ya Huang[1,2], Dengfeng Li[1,2], Qinglei Guo[5], Yuhang Li*[3,6], and Xinge Yu*[1,2,7]

[1]Department of Biomedical Engineering
City University of Hong Kong
Kowloong Tong (Hong Kong)
[2]Hong Kong Center for Cerebra-Cardiovascular Health Engineering
Hong Kong Science Park
New Territories 999077 (Hong Kong)
[3]Institute of Solid Mechanics
Beihang University
Beijing 100191 (China)
[4]China Special Equipment Inspection and Research Institute
Beijing 100029 (China)
[5]Center of Nanoelectronics
School of Microelectronics
Shandong University
Jinan 250100 (China)
[6]Aircraft and Propulsion Laboratory
Ningbo Institute of Technology Beihang University
Ningbo, 315100 (China)
[7]City University of Hong Kong Shenzhen Research Institute
City University of Hong Kong
Shenzhen 518057 (China)

[†]These authors contributed equally: Yiming Liu, Chun Ki Yiu, Zhao Zhao, and Wooyoung Park

E-mail: liyuhang@buaa.edu.cn; xingeyu@cityu.edu.hk

Table S1. Comparison of the state-of-art olfactory interfaces with our work in the aspect of functional module, dimensions, scents, mechanical formats, response time, recovery time, communication method, and applications.

**Functional Module**	**Dimensions (mm)**	**Odor options**	**Mechanical formats**	**Response time**	**Recovery time**	**Communication method**	**Application**	**Ref.**
a surface acoustic wave device and micro-dispensing valves	N/A	1	rigid	10 s	N/A	Wired USB	VR game	(1)
mass flow controllers	N/A	3	rigid	2.5 s	N/A	Wired	olfactory virtual reality for mice	(2)
Commercial atomizer	L=500<br>W=500<br>T=400	3	rigid	5.8 s	N/A	Bluetooth	Improving users' sleep quality	(3)
Commercial atomizer	L=110<br>W=56<br>T=35	1	rigid	6 s	N/A	Wi-Fi	VR game	(4)
BFM based atomizer	N/A	4	rigid	3.1 s	5.16 s	Wired	VR technology	(5)
surface acoustic wave device and micro-dispensing valves	N/A	1	rigid	N/A	N/A	Wired	VR game	(6)
Commercial atomizer	N/A	1	rigid	N/A	N/A	Wired	Olfaction display	(7)
Physical phase change of odorous paraffin wax	N/A	6	rigid and non-wearable	<180 s & >120 s	30	Bluetooth	Olfaction training	(8)
Commercial atomizer	L=67<br>W=60<br>T= 82	6	flexible, wearable	N/A	N/A	Bluetooth	VR game	(9)
Multisensory VR System	N/A	3	rigid	5.7 s	N/A	Wired	VR game	(10)
Direct-Injection	L=55<br>W=15<br>T=40	3	wearable	0.5 s	0.5 s	Wired USB	Olfaction training	(11)
SAW Atomizer	N/A	8	rigid	6 s	N/A	Wired	Olfactory display for multimedia content and virtual reality	(12)
Commercial atomizer	L=150<br>W=150	4	rigid	N/A	N/A	Bluetooth	museum exhibitions	(13)

	D=60							
Physical phase change of odorous paraffin wax by controlling the heating temperature.	L=18<br>W=16<br>T=3	9 basic types	flexible, wearable	1.44 s	40 s for ethanol gas with the peak concentration of 531 ppm.	Bluetooth	VR/AR, human-machine interface, medical treatment, 4D movie, and message delivery.	this work

Table S2. Basic information of the frequently used perfumes.

**Odor name**	**Components**	**Types**	**Pure odor duration time (h)**	**Purchase link**
Lavender	Lavandula angustifolia	Herbal note	19.8	https://item.taobao.com/item.htm?spm=a1z09.2.0.0.2f502e8dt3WiVQ&id=559055572709&_u=o2jk453d28b
Lemon	Chrysophoron, Musk, Grapefruit, Vetiver grass, Petitgrain, Bergamot, Vanilla, Flores aurantia, Cedar.	Fruit note	8.3	https://detail.tmall.com/item_o.htm?abbucket=4&id=644765865889&rn=cca438a41ff37ac4225f73511b702476&spm=a312a.7700824.w4011-23876712565.48.4577b863Kij1q6&sku_properties=1626521:1088826456
Strawberry	Strawberry, Peach, Mandarin orange, Lily of the valley, Violet, Musk.	Fruit note	24.3	https://detail.tmall.com/item_o.htm?abbucket=4&id=646128618010&rn=cca438a41ff37ac4225f73511b702476&spm=a312a.7700824.w4011-23876712565.64.4577b863Kij1q6&sku_properties=1626521:6210120
Minty	Mint, Mentha spicata, Niaouli, Musk. Ambergris.	Minty note	4	https://item.taobao.com/item.htm?spm=a1z09.2.0.0.2f502e8dt3WiVQ&id=634588501729&_u=o2jk453feed
Green tea	Lemon, Agrumi di Sicilia, Chamomile, Green tea, Jasmine tea, White flower, Chrysophoron, Honey, Ambrette seeds.	Woody note	31.5	https://detail.tmall.com/item_o.htm?_u=o2jk453a828&id=656299236516&spm=a1z09.2.0.0.2f502e8dt3WiVQ&skuId=4736630916824
Lilac	Eugenia caryophyllus	Flower note	141.8	https://item.taobao.com/item.htm?spm=a1z09.2.0.0.2f502e8dt3WiVQ&id=578197849564&_u=o2jk453353a
Peach	Citrus, Peach, Lily of the valley, Sandalwood	Fruit note	92.5	https://detail.tmall.com/item_o.htm?_u=p2jk453e642&id=645656358985&spm=a1z09.2.0.0.2f502e8dt3WiVQ&sku_properties=1626521:6210120
Pineapple	Pineapple	Fruit note	9.2	https://detail.tmall.com/item_o.htm?_u=p2jk453f27a&id=645414838669&spm=a1z09.2.0.0.2f502e8dt3WiVQ&sku_properties=1626521:6210120
Orange	Citrus aurantium dulcis	Citrus note	3.9	https://item.taobao.com/item.htm?spm=a1z09.2.0.0.2f502e8dt3WiVQ&id=559145201790&_u=o2jk4536a6f

Table S3. Performance of 30 different odor types used in Fig. 4c at a heating temperature of 240°C.

No.	Odor types	Can it release odor at heating temperature of 60°C through OGs?	Heating temperature	Optical images of the odorour wax at the heating temperature of 280°C
1	ethanol	Yes	78°C	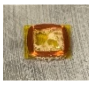
2	pineapple	Yes	240°C	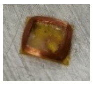
3	grape	Yes	240°C	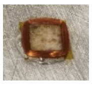
4	mint	Yes	240°C	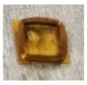
5	rice	Yes	240°C	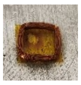
6	cream	Yes	240°C	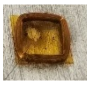
7	gardenia	Yes	240°C	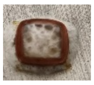
8	Watermelon	Yes	240°C	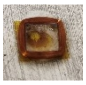
9	vanilla	Yes	240°C	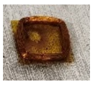
10	coffee milk	Yes	240°C	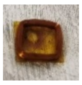
11	candy	Yes	240°C	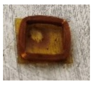
12	Coconut milk	Yes	240°C	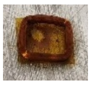
13	Coconut	Yes	240°C	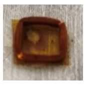
14	milk	Yes	240°C	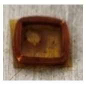
15	peach	Yes	240°C	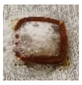
16	pancake	Yes	240°C	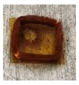
17	orange	Yes	240°C	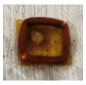
18	green tea	Yes	240°C	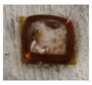

**19**	caramel	**Yes**	**240°C**	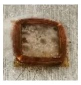
**20**	durian	**Yes**	**240°C**	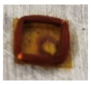
**21**	lemon	**Yes**	**240°C**	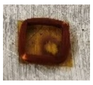
**22**	strawberry	**Yes**	**240°C**	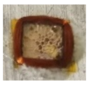
**23**	morning	**Yes**	**240°C**	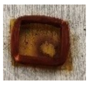
**24**	ginger	**Yes**	**240°C**	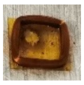
**25**	clary sage	**Yes**	**240°C**	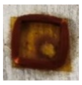
**26**	Rosemary	**Yes**	**240°C**	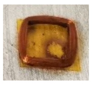
**27**	Lavender	**Yes**	**240°C**	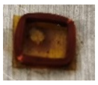
**28**	clove	**Yes**	**240°C**	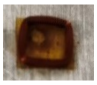
**29**	mojito	**Yes**	**240°C**	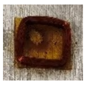
**30**	cake	**Yes**	**240°C**	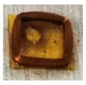

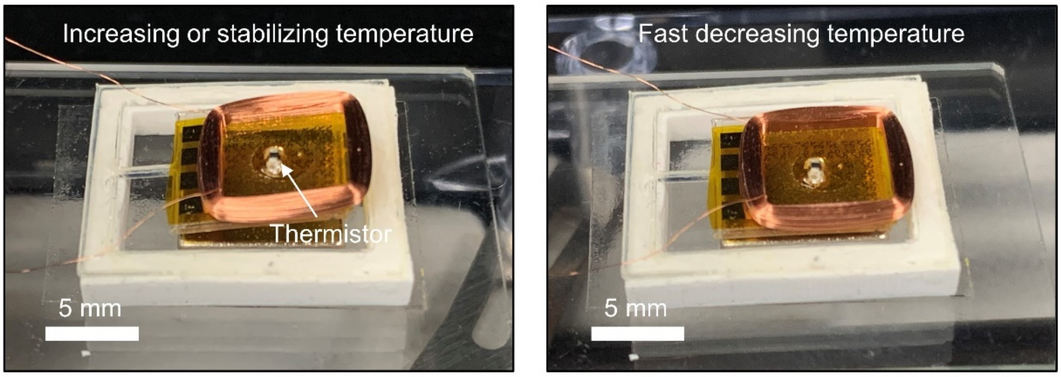

Fig. S1. Optical images of the odor generator under two mechanical conditions, including uplift for increasing or stabilizing temperature, and dropping down for fast decreasing temperature.

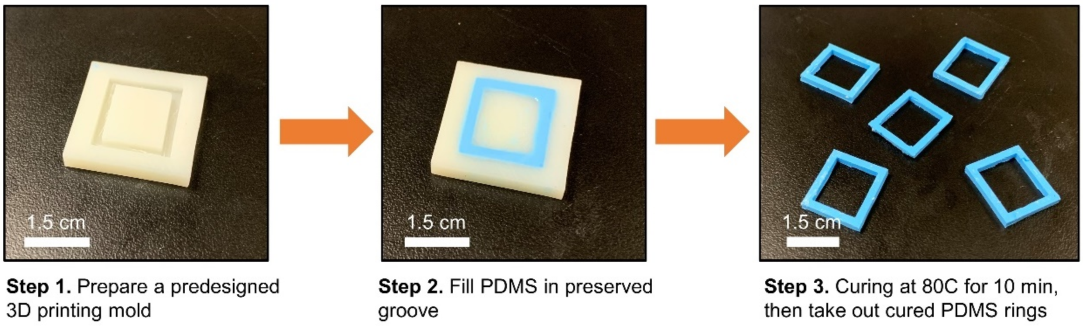

Fig. S2. Fabrication process of the soft ring based on colored PDMS.

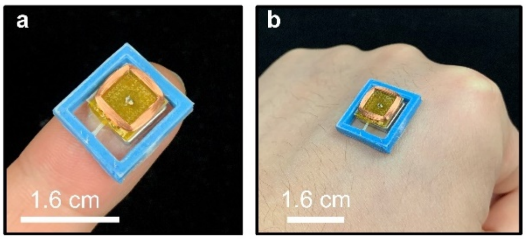

Fig. S3. Optical images of the odor generator mounted onto human finger (a) and hand back (b).

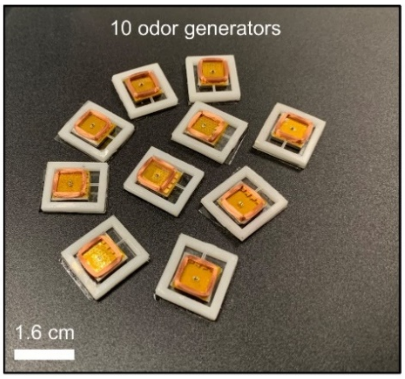

Fig. S4. Optical images of 10 odor generators stacked on the ground.

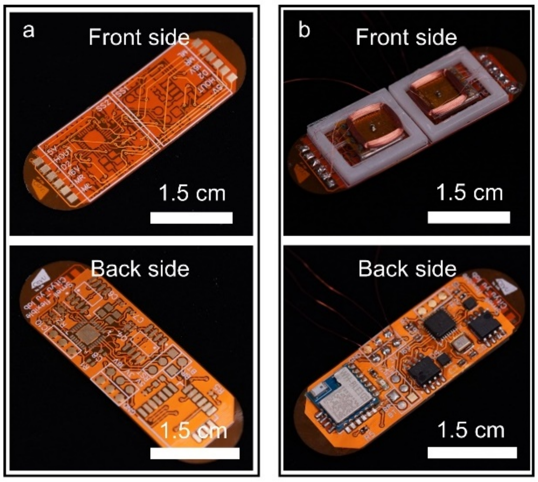

Fig. S5. Optical images of the FPCB design of the device 1 based on 2 OGs.

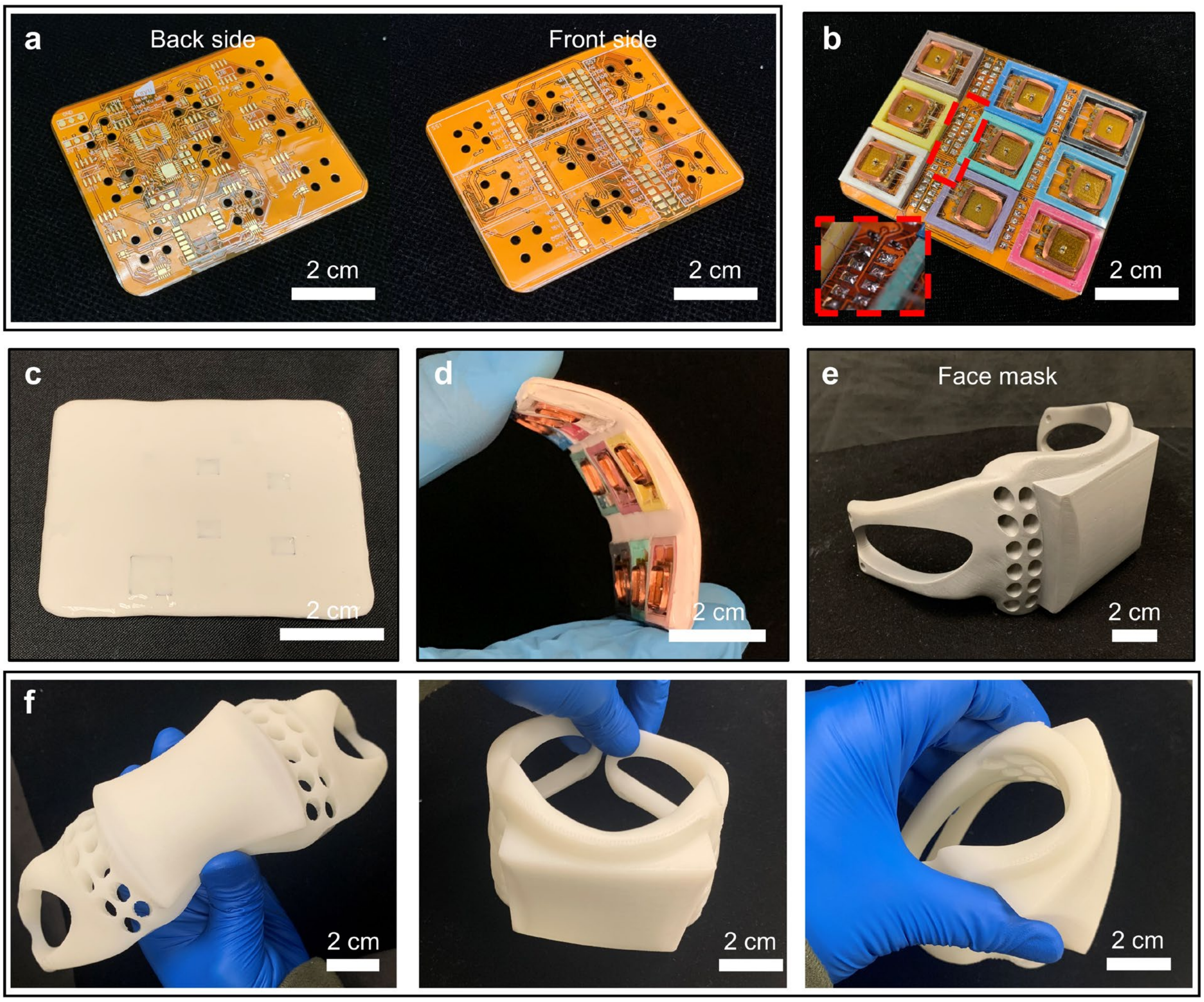

Fig. S6. Illustrations of the device 2 based on 9 OGs. Optical images of the back and front side views of the FPCB **(a)**, 9 OGs soldered onto the FPCB with enlarged details **(b)**, the encapsulated back sides of the FPCB with electrical elements soldered **(c)**, the encapsulated device based on 9 OGs bent at an angle of 30° **(d)**, the self-designed face mask based on soft rubber **(e)**, and the mask under three bending deformations **(f)**.

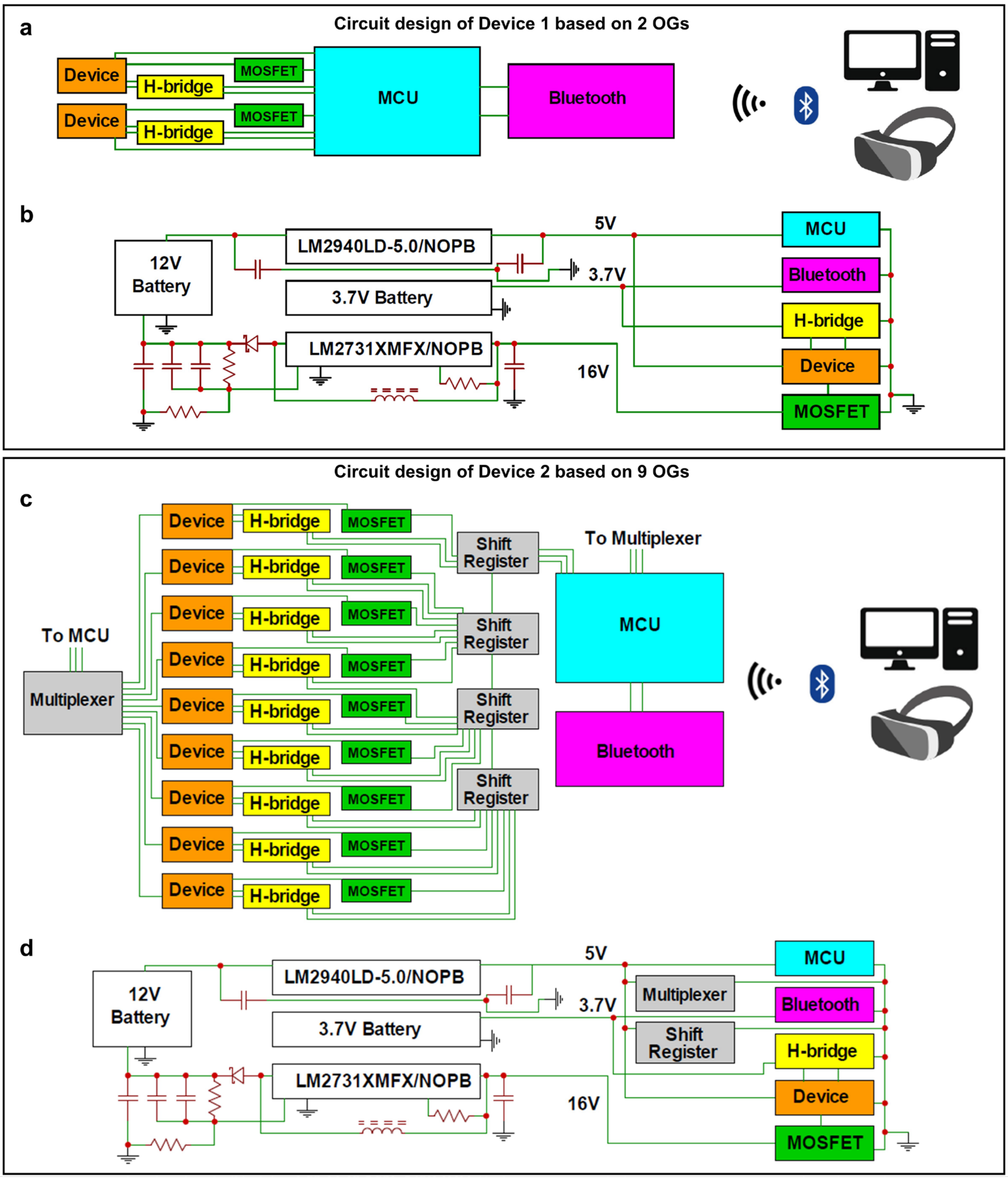

Fig. S7. Circuit design of the two olfaction interfaces. Detailed circuit design of the device 1 **(a, b)** and device 2 **(c, d)**.

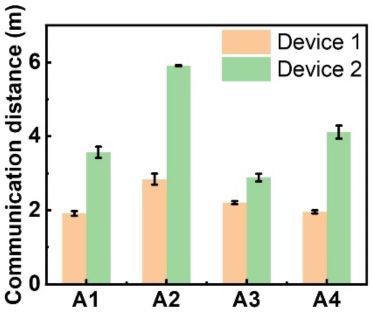

Fig. S8. The largest transmission distance between the two olfaction interfaces and the paired Bluetooth receiver as mounting the olfaction interfaces onto human face with four facing modes to the receiver, including face to one direction in parallel (A1), face to each other (A2), face to one direction in a line (A3), and face perpendicularly to another one (A4). The error bars denote the standard deviation.

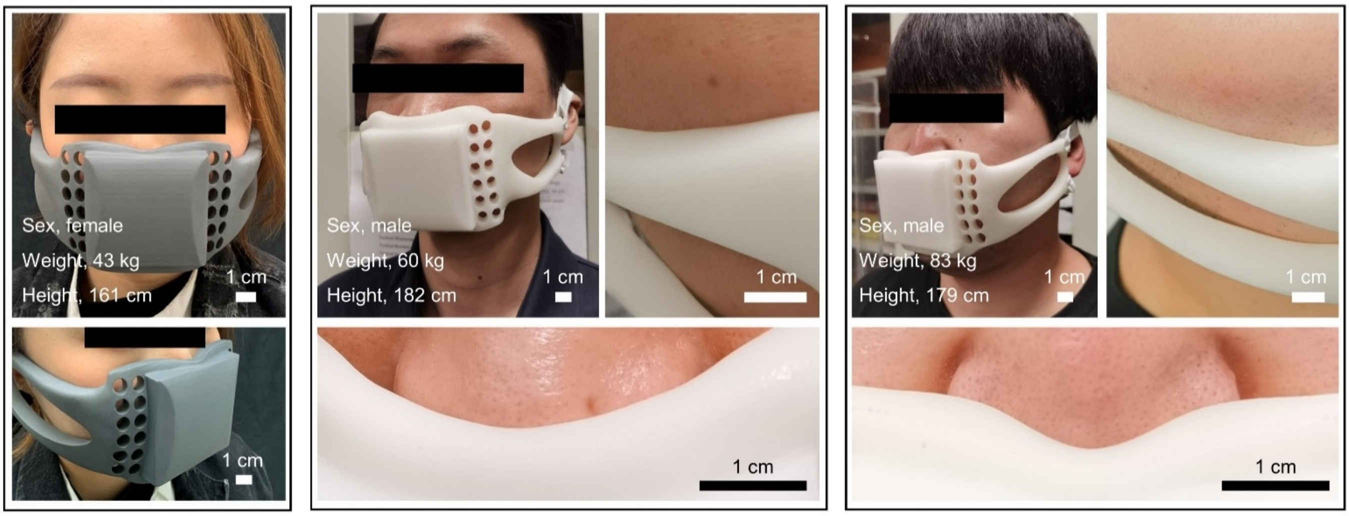

Fig. S9. Optical images of users wearing the Device 2, whose basic physical information have been provided. The flexible face mask substrate enables the wide applications for various users.

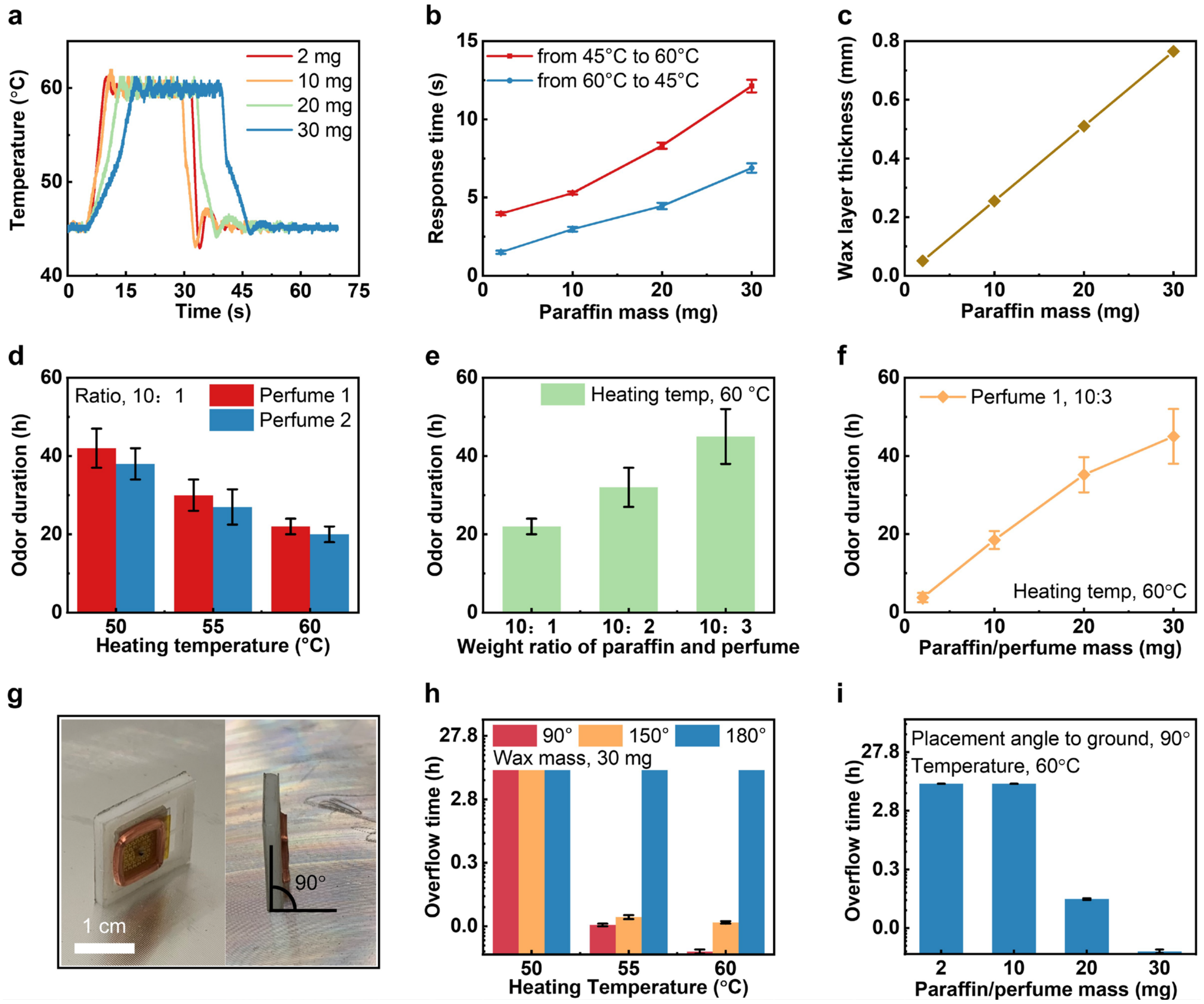

Fig. S10. Extended electrical properties of the odor generator. a, b, c Temperature response of the OG with different odorous paraffin wax mass ranging from 2 mg to 30 mg. **d** Odor duration time of the OG with two different perfume mixed paraffin wax at a constant wax: perfume mass ratio of 10: 1 (odorous wax mass, 30 mg). **e** Odor duration time of the OG with different mass ratios at a constant mass of 30 mg. **f** Odor duration time of the OG with different paraffin was mass ranging from 2 mg to 30 mg at a constant mass ratio of 10: 3. **g, h, i** Overflow time of the melting paraffin wax as the OG is placed onto a flat plane with an intersection angle ranging from 90° to 180°. In this figure, all error bars denote the standard deviation.

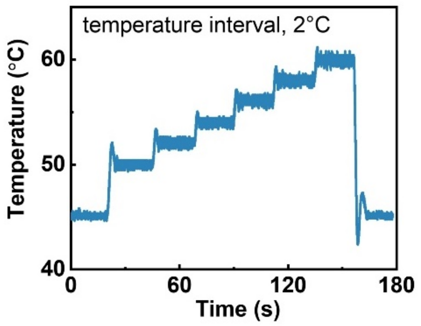

Fig. S11. Controlled temperature of the heating electrode increasing from 45 °C to 60 °C in a constant interval of 2 °C.

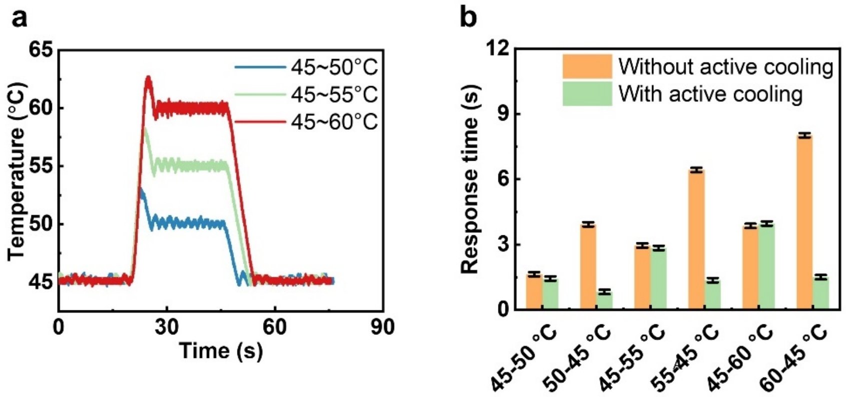

Fig. S12. Temperature response of the heating electrode in air without the mechanical structures for fast dropping temperature (a) and corresponding response time for increasing and decreasing temperature of the heating electrode (b). The error bars denote the standard deviation.

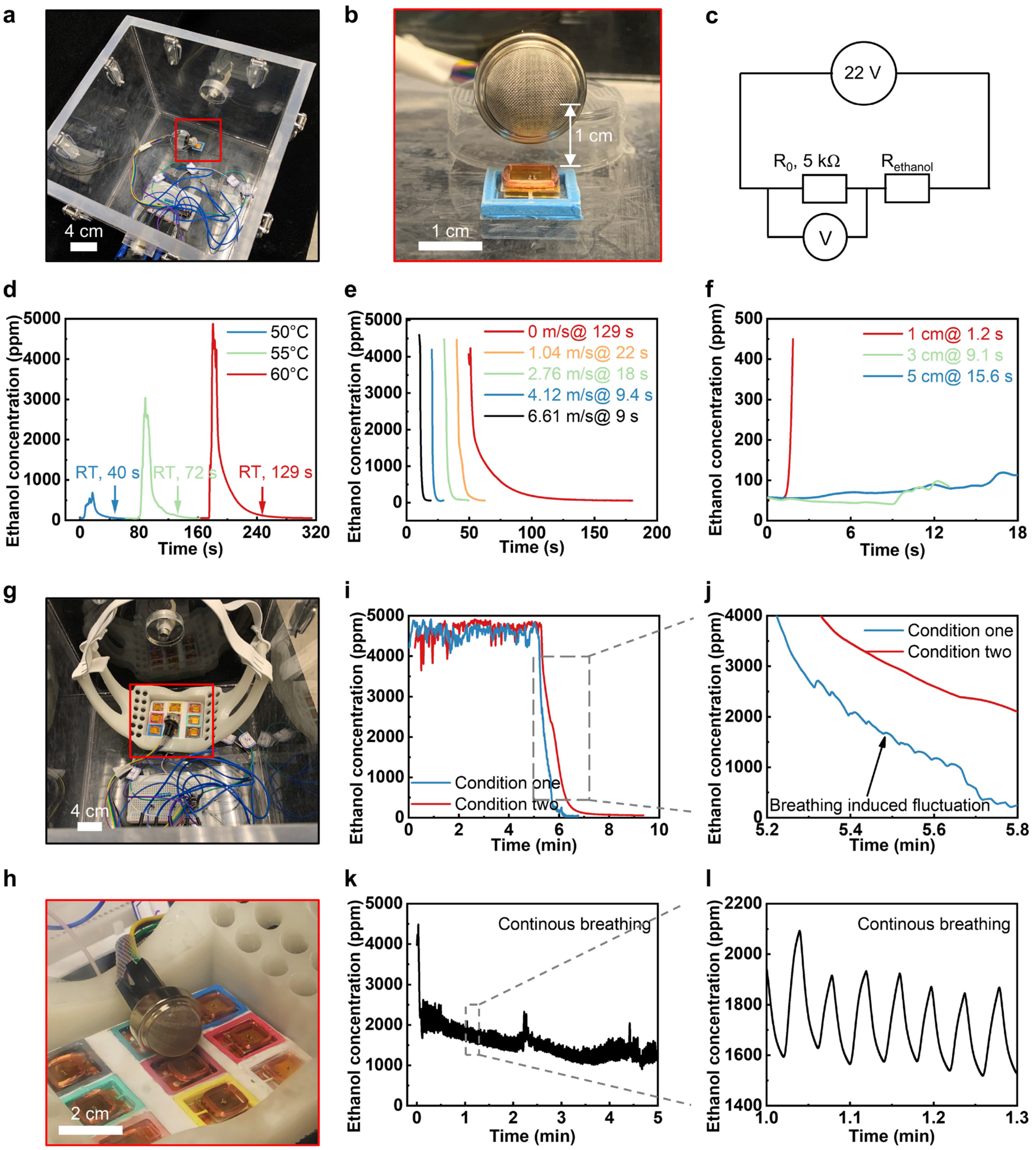

Fig. S13. Ethanol concentration performance of the odor generators. **a, b, c** Ethanol concentration testing setup, where the test is placed insides an open box for minimizing the ambient wind interference to OGs. During the testing, a commercial resistance-variation-based ethanol sensor placed 1 cm above a working OG for low response time to generated ethanol around the OG. **d** Ethanol concentration generated by OG incorporating the paraffin/ethanol (mass ratio, 10:3) with a controlled temperature switching between 45°C to 50°C, 55°C, and 60°C, respectively, corresponding to the increasing ethanol concentration peak values. **e** Ambient wind effects on the ethanol concentration dissipation rate, here an operating OG with a heating temperature of 60°C are suddenly shut down to cut off ethanol generation, and the wind is blown above the OG parallelly. **f** Delay time of a working OG as a function of the distance between the generator and ethanol sensor, which is induced by the ethanol diffusion rate. **g, h, i, j, k, l** Ethanol remaining time test in Device 2

with three testing conditions. Condition one is that experimenter continuously smell the generated ethanol after the OG with paraffin/ethanol embedded works for 5 min at a constant heating temperature of 60°C when he is wearing the Device 2, and Condition two is that the ethanol sensor monitors the ethanol concentration after the OG works for 5 min in an open box (i, j). Condition three is that the ethanol sensor continuously monitors gas concentration as the experimenter is wearing the Device 2 (k, l).

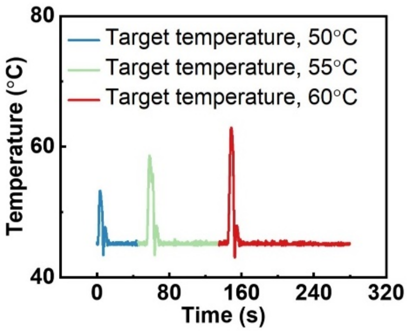

Fig. S14. Electrical response of a working OG with the heating temperature switching between 45 °C and 50 °C, 55 °C, and 60°C with the generated ethanol concentration shown in Supplementary Fig. 13d.

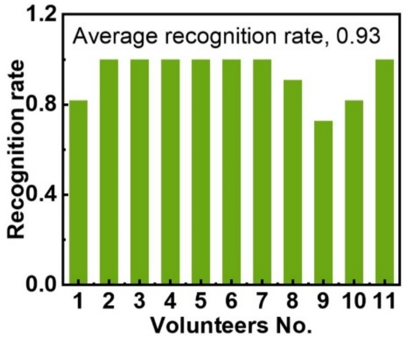

Fig. S15.A volunteer testing showing the volunteers' odor recognition rate when they are wearing the Device 2 with 9 different perfume-based odors (see details in Characterization). Here, the temperature of each OGs will be increased from 45 °C to 60 °C at an interval of 5 °C, where each temperature will last 1 min before going up. The, the volunteers will be asked if they could sense the enhanced odor concentration.

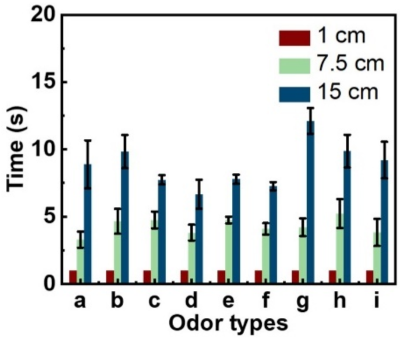

Fig. S16. Delay time as a function of the distance between OGs and user's nose for 9 odor types. Here, a, b, c, d, e, f, g, h, and i stand for lavender, orange, pineapple, green tea, lemon, peach, strawberry, minty, and lilac. The error bars denote the standard deviation.

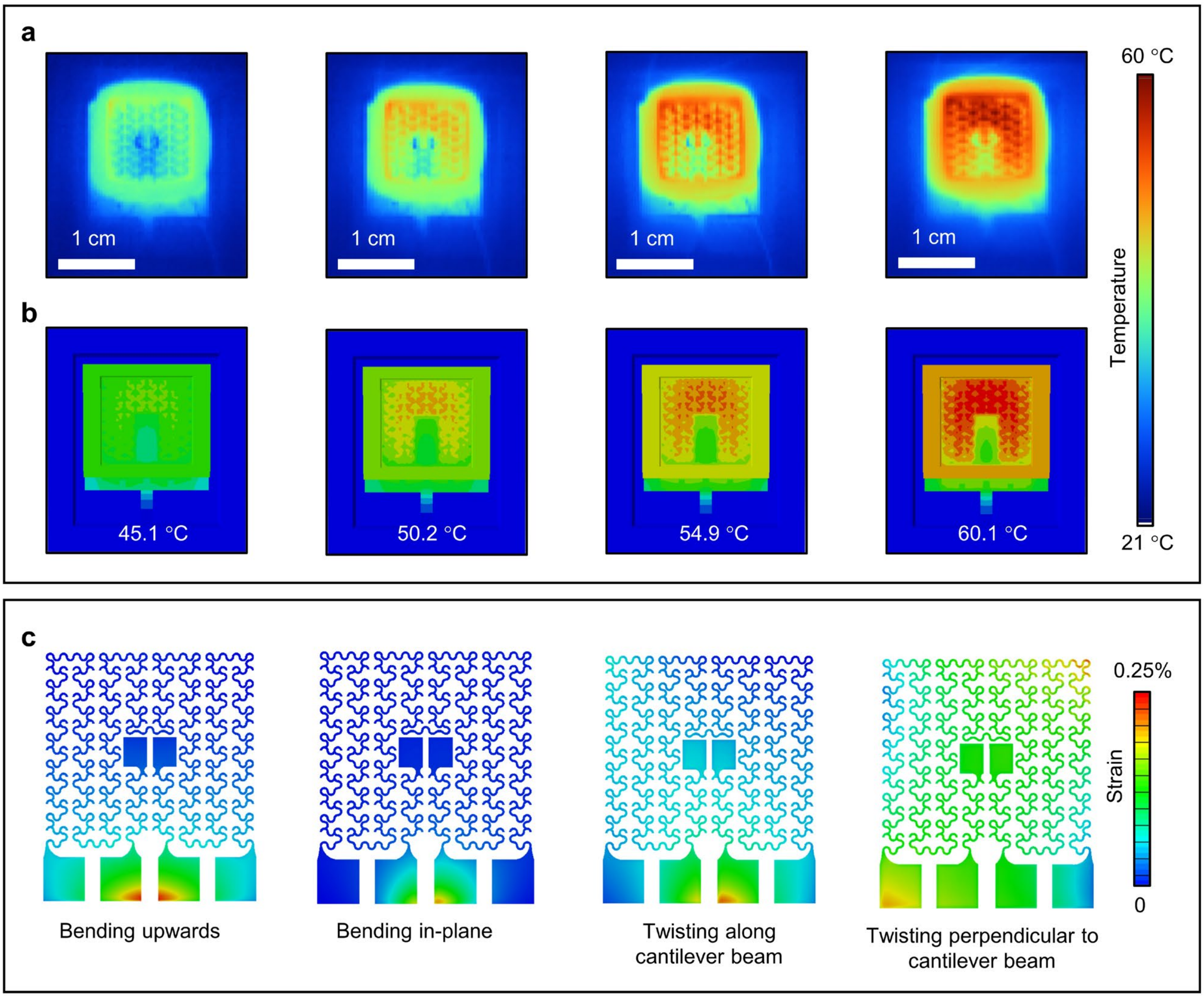

Fig. S17. a, b thermal stimulation results and corresponding real condition of the OG with a stabilizing temperature of 45°C, 50°C, 55°C, and 60°C. **c** Mechanical stimulation result of the OG heating electrode under four mechanical deformations.

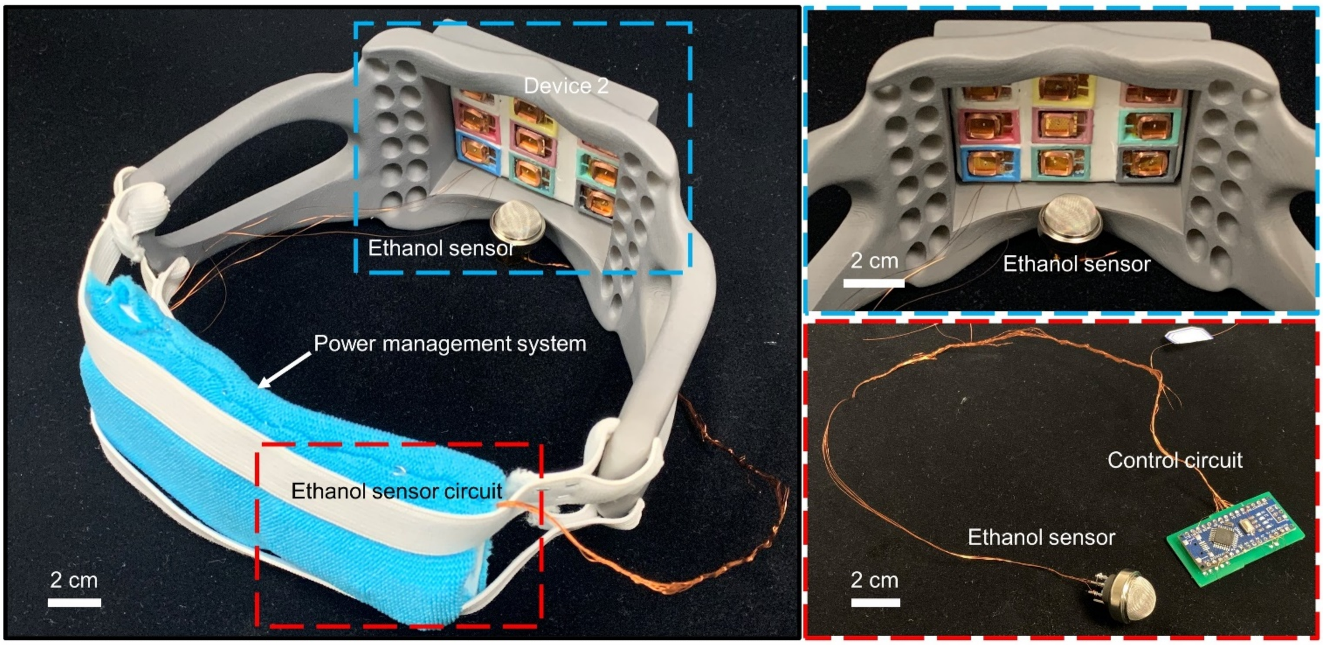

Fig. S18. Optical images of the Device 2 with the commercial ethanol sensor integrated.

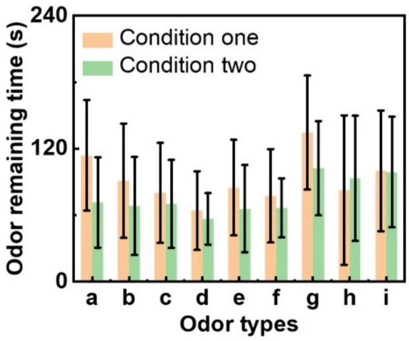

Fig. S19.A volunteer testing showing the odor remaining time in Device 2. Here, all volunteers will be asked to smell the remaining odors in the Device 2, and record the time when the odors disappear. There are two testing conditions. Condition One is that volunteers continuously smell the odors when wearing the Device 2. Condition Two is that the volunteers come to smell the odors of the face mask every 10 s. Before volunteer smells the Device 2, the OG will generate the odor at a constant heating temperature of 60 °C in the face mask for 5 min. The error bars denote the standard deviation.

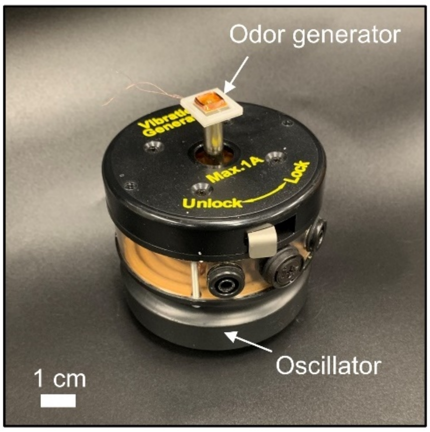

Fig. S20. Optical images of the odor generator mounted onto a vibration platform with adjustable

vibrication frequency and applitumde.

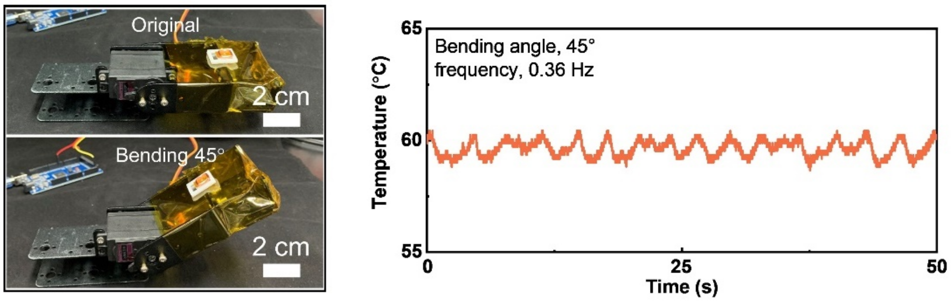

Fig. S21. Optical images of the odor generator mounted on a programmable bending platform (a), and the temperature response of the heating electrode with a target temperature of 60 °C at a constant bending angle and frequency of 45° and 0.36 Hz (b).

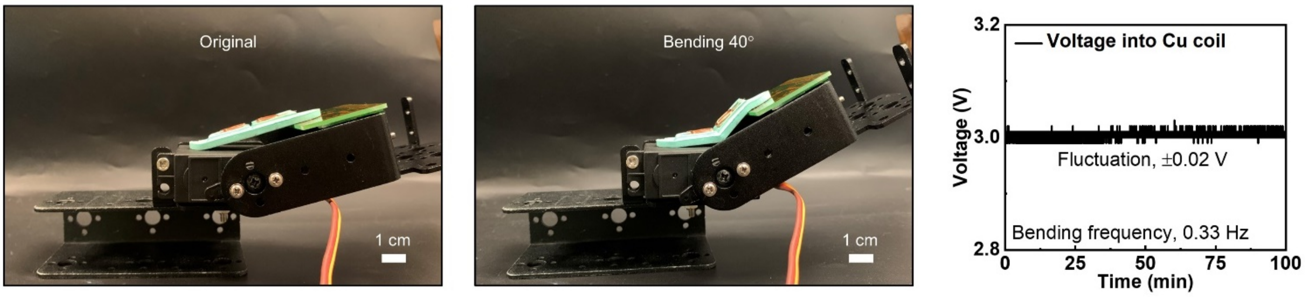

Fig. S22. Optical images and electrical response of the Device 1 at a bending angle of 40° for over 2000 cycles. Here, the electrical signal is the voltage into the electromagnetic coils of two OGs in Device 1, which is wirelessly read by a paired receiver. The stable voltage input demonstrates the normal operation of the Device 1 during continuous bending.

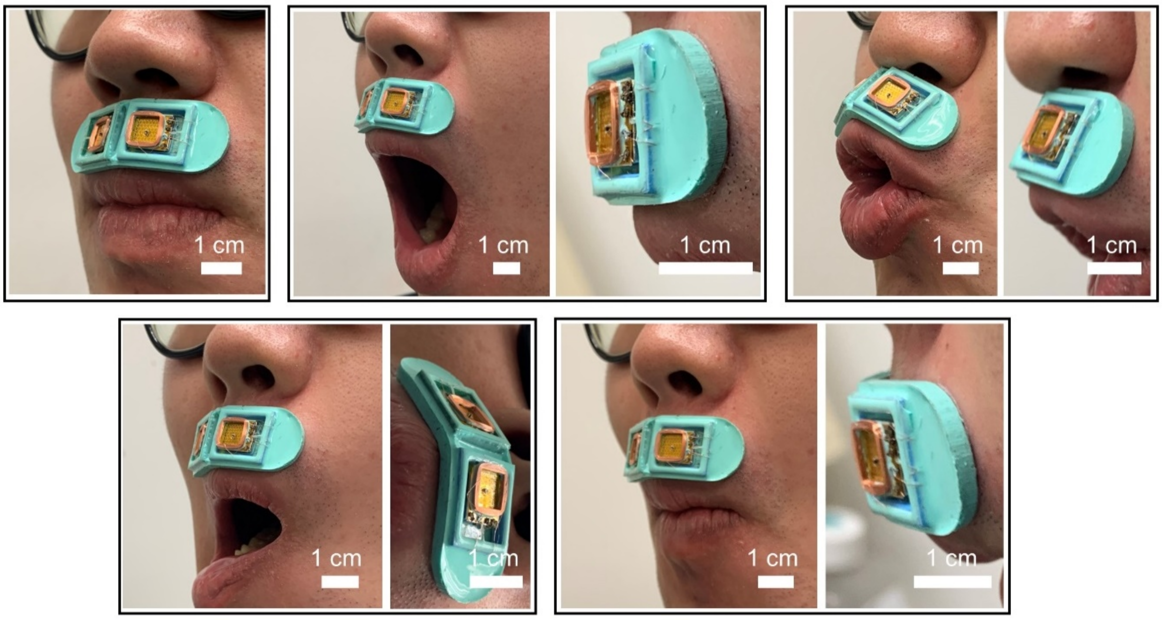

Fig. S23. Optical images of Device 1 mounted onto human upper lip. As users do various mouth motions,

the Device 1 can be still tightly mounted onto the skin.

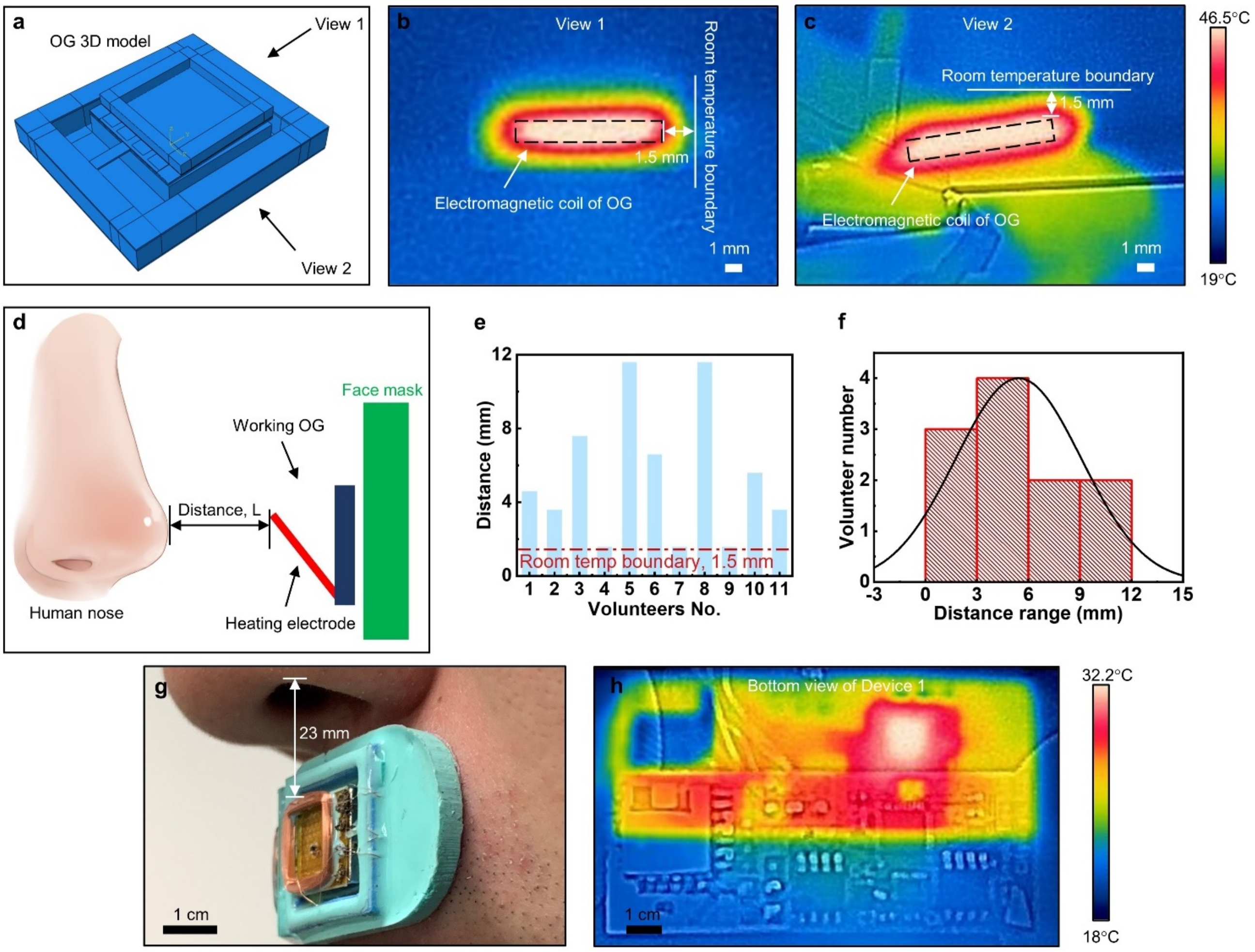

Fig. S24. Safety operation of Device 2 and Device 1. (a) A OG 3D model built up in software ABAQUS (Analysis User's Manual 6.14). (b, c) Two views of thermal distribution of a working OG with the working temperature stabilized around 60 °C, where the working temperature is the one around the thermistor for melting odorous wax insides OGs. (d) Schematic diagram of the inside layout of Device 2 when users are wearing the Device 2. Here, the distance between users' nose and working OGs decides the safety of Device 2 during operation. (e, f) 11 volunteers' distance values between volunteers' nose and working OGs, including 4 males and 7 females. All volunteers can safely wear the Device 2 during its operation as the minimum distance value is 2 mm, where the air temperature is room temperature. (g) Optical image of a user wearing the Device 1 on his upper lip with a value (23 mm) showing the distance between the OG and the nearby human nose. (h) Thermal distribution of the Device 1 bottom side with the working temperatures of two OGs stabilized around 60 °C. The temperature peak is 32.2 °C, demonstrating that the Device 1 has no potential risk to the attached human skin.

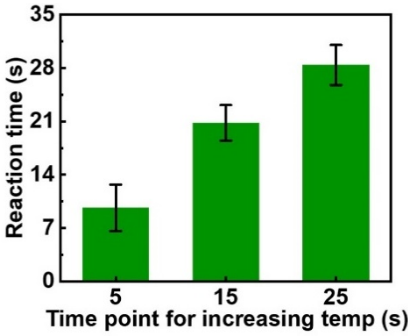

Fig. S25. A volunteer testing showing the human reaction to the fast temperature variation of OGs with two odor types embedded when they are wearing Device 1 for testing. During the test, the Device 1 will be programmed to increase temperatures of OGs from 45 °C to 50 °C at 5 s, from 50 °C to 55°C at 15 s, from 55 °C to 60 °C at 25 s, then lasting 1 min until shutting down Device 1. Once the volunteers can sense the enhanced odor concentration, the time point will be recorded meanwhile. Two different odor types for two OGs in Device 1 are adopted, including lavender and lilac. The error bars denote the standard deviation.

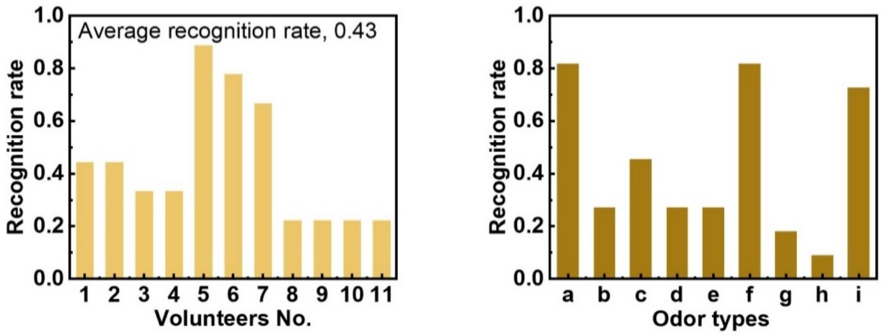

Fig. S26. A volunteer testing showing the recognition rate of volunteers in smelling the OGs at room temperature when they are the Device 1. Here, a, b, c, d, e, f, g, h, and i stand for lavender, orange, pineapple, green tea, lemon, peach, strawberry, minty, and lilac.

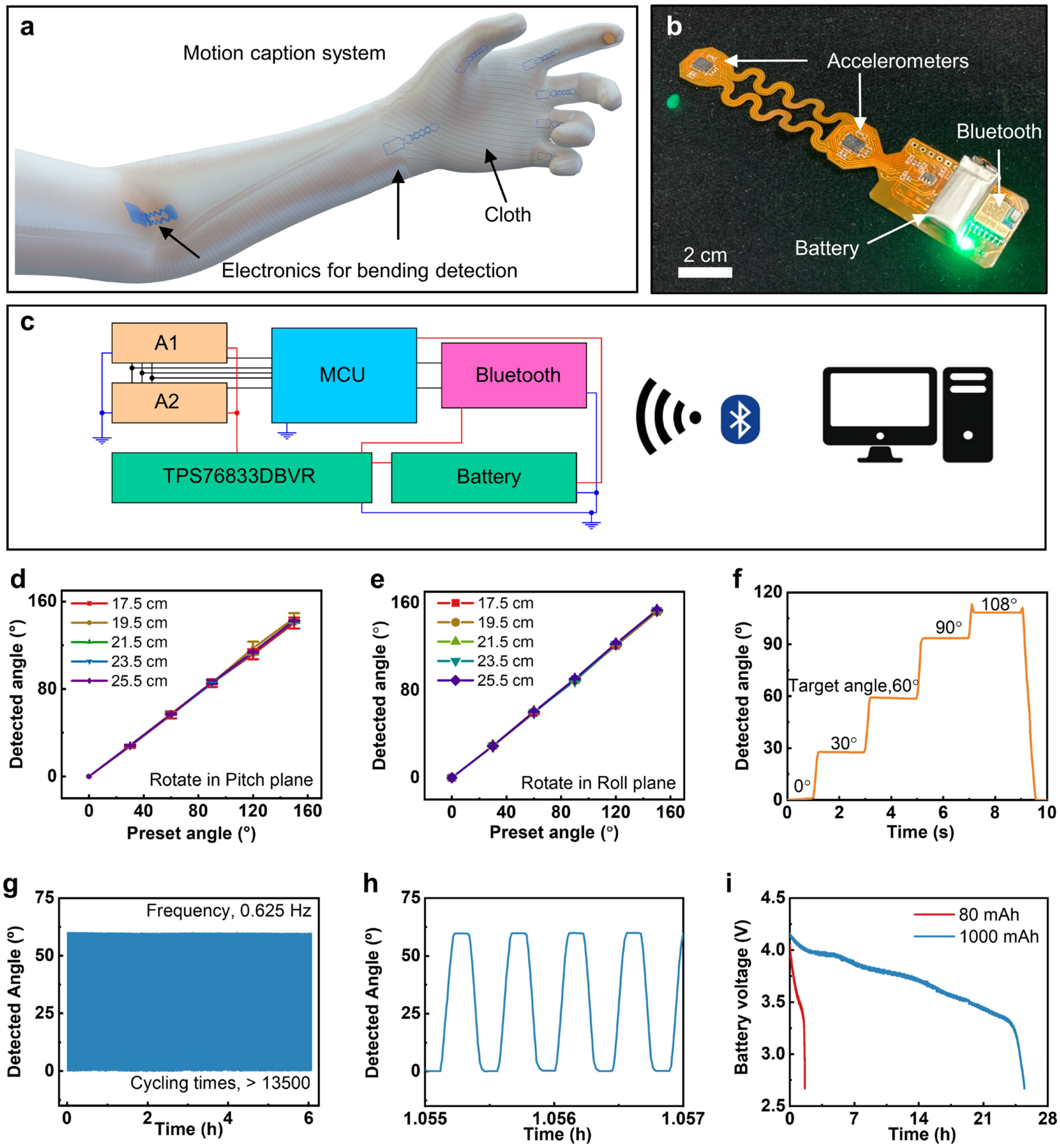

Fig. S27. Electrical characteristics of the motion caption system. a Schematic diagram of the motion caption system with cloth integrated. **b** Optical image of the wireless motion caption electronics based on accelerometers. **c** Circuit design of the programmable motion caption electronics. **d, e** Electrical response of the motion caption electronics bent to different angles in Pitch and Roll planes with different distance between two accelerometers. Here, the error bars denote the standard deviation. **f** Electrical response of the motion caption electronics bent from 0 to 108° step by step in a Pitch plane. **g, h** Stability testing of the motion caption electronics continuously bent from 0 to 60° for over 13500 cycles. **i** Operation time of the motion caption electronics with the voltage output of the two batteries (energy capacities, 80 mAh and 1000 mAh) dropping from 4 V to 2.5 V.

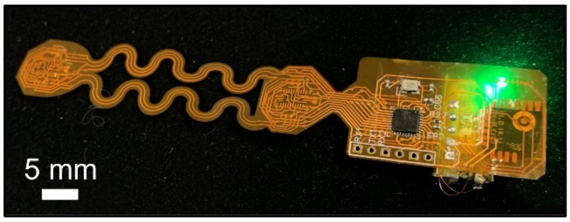

Fig. S28. Optical images of the motion caption electronics back side with a MCU soldered.

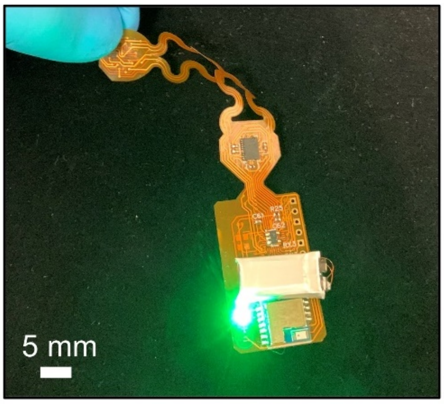

Fig. S29. Optical images of the motion caption electronics under a twisted deformation.

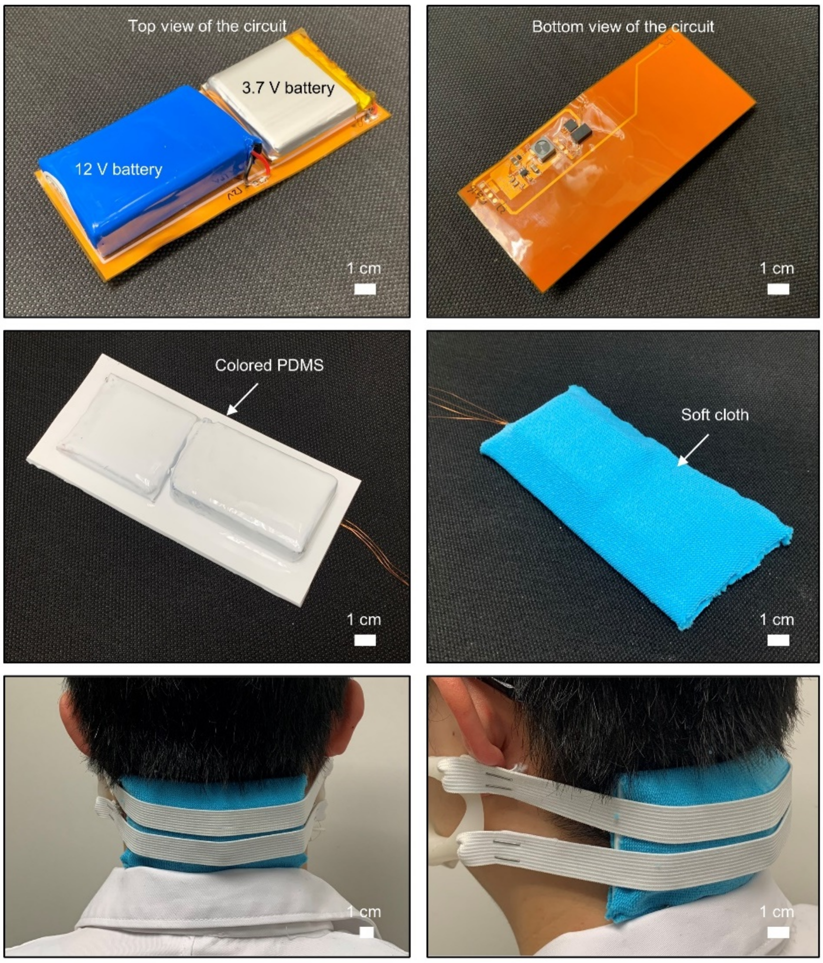

Fig. S30. Optical images of the flexible power management system, where there are two batteries, including a 3.7 V battery (2000 mAh) and 12 V battery (1800 mAh).

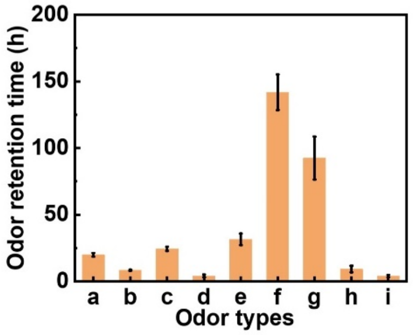

Fig. S31. The retention time of 9 pure perfumes adopted in Figs. 4a, b, e. Here, a, b, c, d, e, f, g, h, and i stand for lavender, orange, pineapple, green tea, lemon, peach, strawberry, minty, and lilac. The error bars denote the standard deviation.

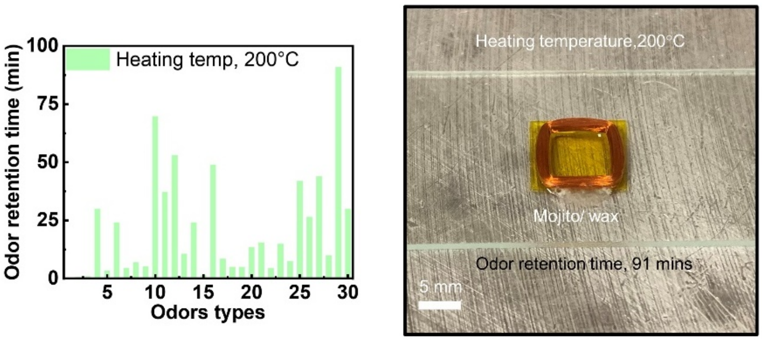

Fig. S32. The retention time and optical images of 30 odor types adopted in Fig. 4c. From No. 1 to No. 30, the odor types are ethanol, pineapple, grape, mint, rice, cream, gardenia, watermelon, vanilla, coffee milk, candy, coconut milk, coconut, milk, peach, pancake, orange, green tea, caramel, durian, lemon, strawberry, morning, ginger, clary sage, rosemary, lavender, clove, mojito, and cake, respectively. Among the 30 odor types, some odor types could continuously release smell at a high heating temperature of 200°C for over 1 h, such as mojito and coffee milk. There is no boiling phenomenon observed for the mojito odor type. It is concluded that the mojito odor type is a low-volatile one with boiling point higher than 200°C. It has been proven that volunteers could obviously sense the mojito odor generated by Device 2 shown in Fig. 4c, therefore, it is proven that the low-volatile compound with boiling point higher than 200°C can be generated by adopting Device 2.

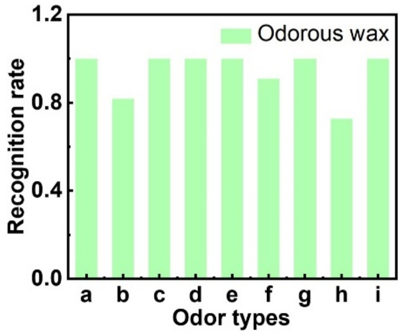

Fig. S33. An volunteer test showing the 11 volunteers' recognition rates to the 9 different odorous wax samples, which is prepared three weeks ago, then exposuring in air at room temperature with the relative humidity of 60%. During the testing, all volunteers are required to wear the Device 2 with the 9 different wax samples in each OGs. Here, OGs heating temperature is 60 °C. For odor types, a, b, c, d, e, f, g, h, and i stand for lavender, orange, pineapple, green tea, lemon, peach, strawberry, minty, and lilac.

Reference

1 S. Kato, T. Nakamoto, in 2018 IEEE Conference on Virtual Reality and 3D User Interfaces (VR). (IEEE, 2018), pp. 597-598.

2 B. A. Radvansky, D. A. Dombeck, An olfactory virtual reality system for mice. Nature communications 9, 1-14 (2018).

3. J. Amores, M. Dotan, P. Maes, Development and Study of Ezzence: A Modular Scent Wearable to Improve Wellbeing in Home Sleep Environments. Frontiers in psychology, 550 (2022).

4. M. de Paiva Guimarães, J. M. Martins, D. R. C. Dias, R. d. F. R. Guimarães, B. B. Gnecco, An olfactory display for virtual reality glasses. Multimedia Systems, 1-11 (2022).

5. P. Yang et al., Self-powered virtual olfactory generation system based on bionic fibrous membrane and electrostatic field accelerated evaporation. EcoMat, e12298 (2022).

6. S. Kato, T. Nakamoto, in 2019 IEEE International Symposium on Olfaction and Electronic Nose (ISOEN). (IEEE, 2019), pp. 1-3.

7. Y. Wang, J. Amores, P. Maes, in Proceedings of the 2020 CHI Conference on Human Factors in Computing Systems. (2020), pp. 1-9.

8. A. Tiele, S. Menon, J. A. Covington, Development of a Thermal-Based Olfactory Display for Aroma Sensory Training. IEEE Sensors Journal 20, 631-636 (2019).

9. S. Zou, X. Hu, Y. Ban, S. i. Warisawa, in 2022 IEEE Conference on Virtual Reality and 3D User Interfaces (VR). (IEEE, 2022), pp. 474-482.

10. A. Bahremand et al., in 2022 IEEE Conference on Virtual Reality and 3D User Interfaces (VR). (IEEE, 2022), pp. 241-249.

11. T. Yamada, S. Yokoyama, T. Tanikawa, K. Hirota, M. Hirose, in IEEE Virtual Reality Conference (VR 2006). (IEEE, 2006), pp. 199-206.

12. T. Nakamoto, S. Ito, S. Kato, G. P. Qi, Multicomponent olfactory display using solenoid valves and SAW atomizer and its blending-capability evaluation. IEEE Sensors Journal 18, 5213-5218 (2018).

13. M. Bordegoni, M. Carulli, S. Bader, in 2019 IEEE International Symposium on Olfaction and Electronic Nose (ISOEN). (IEEE, 2019), pp. 1-3.
