# Supplementary material for: Soft, miniaturized, wireless olfactory interface for virtual reality
Source: Nat Commun. 2023 May 9;14:2297. doi: 10.1038/s41467-023-37678-4 (PMC10169775; doi:10.1038/s41467-023-37678-4)
Supplement: Supplementary file 3 — Description of Additional Supplementary Files Document [file 41467_2023_37678_MOESM3_ESM.pdf]

Description of Additional Supplementary Information Files Document

Video name: Supplementary Movie 1

Description: This movie demonstrates the accurate heating temperature control of the OG through the self-developed control panel.

Video name: Supplementary Movie 2

Description: This movie demonstrates the application of the Device 1 in the 4D movie watching.

Video name: Supplementary Movie 3

Description: This movie demonstrates the application of the Device 2 in a VR game.
