# Supplementary material for: Soft, miniaturized, wireless olfactory interface for virtual reality
Source: Nat Commun. 2023 May 9;14:2297. doi: 10.1038/s41467-023-37678-4 (PMC10169775; doi:10.1038/s41467-023-37678-4)
Supplement: Supplementary file 7 — Reporting Summary [file 41467_2023_37678_MOESM7_ESM.pdf]

Corresponding author(s): Xinge Yu and Yuhang Li

Last updated by author(s): Mar 21, 2023

Reporting Summary

Nature Portfolio wishes to improve the reproducibility of the work that we publish. This form provides structure for consistency and transparency in reporting. For further information on Nature Portfolio policies, see our Editorial Policies and the Editorial Policy Checklist.

Statistics

For all statistical analyses, confirm that the following items are present in the figure legend, table legend, main text, or Methods section.

n/a	Confirmed	
☐	☒	The exact sample size (*n*) for each experimental group/condition, given as a discrete number and unit of measurement
☐	☒	A statement on whether measurements were taken from distinct samples or whether the same sample was measured repeatedly
☒	☐	The statistical test(s) used AND whether they are one- or two-sided<br>*Only common tests should be described solely by name; describe more complex techniques in the Methods section.*
☒	☐	A description of all covariates tested
☒	☐	A description of any assumptions or corrections, such as tests of normality and adjustment for multiple comparisons
☐	☒	A full description of the statistical parameters including central tendency (e.g. means) or other basic estimates (e.g. regression coefficient) AND variation (e.g. standard deviation) or associated estimates of uncertainty (e.g. confidence intervals)
☒	☐	For null hypothesis testing, the test statistic (e.g. *F*, *t*, *r*) with confidence intervals, effect sizes, degrees of freedom and *P* value noted<br>*Give P values as exact values whenever suitable.*
☒	☐	For Bayesian analysis, information on the choice of priors and Markov chain Monte Carlo settings
☒	☐	For hierarchical and complex designs, identification of the appropriate level for tests and full reporting of outcomes
☒	☐	Estimates of effect sizes (e.g. Cohen's *d*, Pearson's *r*), indicating how they were calculated

Our web collection on statistics for biologists contains articles on many of the points above.

Software and code

Policy information about availability of computer code

Data collection	Powerlab by AD Instrument, DAQ6510 data acquisition/multi-meter system, Self-developed control panel for resistance measurement.
Data analysis	Origin 2018, MATLAB R2017b.

For manuscripts utilizing custom algorithms or software that are central to the research but not yet described in published literature, software must be made available to editors and reviewers. We strongly encourage code deposition in a community repository (e.g. GitHub). See the Nature Portfolio guidelines for submitting code & software for further information.

Data

Policy information about availability of data

All manuscripts must include a data availability statement. This statement should provide the following information, where applicable:

- Accession codes, unique identifiers, or web links for publicly available datasets
- A description of any restrictions on data availability
- For clinical datasets or third party data, please ensure that the statement adheres to our policy

All data is contained within the manuscript. Requests can be made to the corresponding author for access to the raw data.

Human research participants

Policy information about studies involving human research participants and Sex and Gender in Research.

Reporting on sex and gender	In our study, we recruited 11 volunteers (4 males and 7 females) for volunteer experiments. Gender was not a factor to be considered in the experiment design. In this study, all volunteer sensory tests we conducted are to investigate the odor generation performance of our olfaction systems, where gender is not a variable that affects the results of the experiment. In addition, each volunteer has been paid 200 HKD for the volunteer tests. Before all the volunteer tests, all volunteers' informed consents have been obtained.
Population characteristics	11 volunteers with 4 males and 7 females. All volunteers are healthy in a normal emotion range.
Recruitment	We posted recruitment flyers in the university to recruit volunteers. The volunteers contacted us by themselves.
Ethics oversight	City University of Hong Kong, Hong Kong, China

Note that full information on the approval of the study protocol must also be provided in the manuscript.

Field-specific reporting

Please select the one below that is the best fit for your research. If you are not sure, read the appropriate sections before making your selection.

☒ Life sciences ☐ Behavioural & social sciences ☐ Ecological, evolutionary & environmental sciences

For a reference copy of the document with all sections, see nature.com/documents/nr-reporting-summary-flat.pdf

Life sciences study design

All studies must disclose on these points even when the disclosure is negative.

Sample size	11 volunteers with 4 males and 7 females. All volunteers are healthy in a normal emotion range.
Data exclusions	No data is excluded from the analyses.
Replication	All of the measurements were taken for multiple times. The data at each test location were sampled for multiple times (more than 3 times) during the measurements. From the measured results, the data showed high similarity, and the reproducibility of all kinds of measurements were good in this study.
Randomization	The human participants were recruited from outside the group by posting flyers.
Blinding	All human participants were only told the experimental process, but didn't know the test results. All human participants did not involve data processing.

Reporting for specific materials, systems and methods

We require information from authors about some types of materials, experimental systems and methods used in many studies. Here, indicate whether each material, system or method listed is relevant to your study. If you are not sure if a list item applies to your research, read the appropriate section before selecting a response.

Materials & experimental systems

n/a	Involved in the study	
☒	☐	Antibodies
☒	☐	Eukaryotic cell lines
☒	☐	Palaeontology and archaeology
☒	☐	Animals and other organisms
☒	☐	Clinical data
☒	☐	Dual use research of concern

Methods

n/a	Involved in the study	
☒	☐	ChIP-seq
☒	☐	Flow cytometry
☒	☐	MRI-based neuroimaging

March 2021
